# Reporting Guidelines for Music-based Interventions: an update and validation study

**DOI:** 10.3389/fpsyg.2025.1551920

**Published:** 2025-06-02

**Authors:** Sheri L. Robb, Stacey Springs, Emmeline Edwards, Tasha L. Golden, Julene K. Johnson, Debra S. Burns, Melita Belgrave, Joke Bradt, Christian Gold, Assal Habibi, John R. Iversen, Miriam Lense, Jessica A. MacLean, Susan M. Perkins

**Affiliations:** ^1^School of Nursing and School of Medicine, Indiana University, Indianapolis, IN, United States; ^2^Faculty of Arts and Sciences, Harvard University, Cambridge, MA, United States; ^3^National Center for Complementary and Integrative Health, Bethesda, MD, United States; ^4^Center for Arts in Medicine, University of Florida, Gainesville, FL, United States; ^5^Institute for Health & Aging, University of California San Francisco, San Francisco, CA, United States; ^6^College of Communication and Fine Arts, University of Memphis, Memphis, TN, United States; ^7^School of Music, Dance, and Theatre, Arizona State University, Tempe, AZ, United States; ^8^Department of Creative Arts Therapies, Drexel University, Philadelphia, PA, United States; ^9^NORCE Norwegian Research Centre AS, Bergen, Norway; ^10^Grieg Academy Department of Music, University of Bergan, Bergen, Norway; ^11^Department of Clinical and Health Psychology, Faculty of Psychology, University of Vienna, Vienna, Austria; ^12^Brain and Creativity Institute, University of Southern California, Los Angeles, CA, United States; ^13^Department of Psychology, Neuroscience and Behaviour, McMaster University, Hamilton, ON, Canada; ^14^Vanderbilt University Medical Center, Nashville, TN, United States; ^15^Department of Speech, Language, Hearing Sciences and Program in Neuroscience, Indiana University, Bloomington, IN, United States; ^16^School of Medicine and Richard M. Fairbanks School of Public Health, Indiana University, Indianapolis, IN, United States

**Keywords:** reporting guidelines, music, music therapy, intervention, reporting quality

## Abstract

**Background:**

Detailed intervention reporting is essential to interpretation, replication, and translation of music-based interventions (MBIs). The 2011 *Reporting Guidelines for Music-Based Interventions* were developed to improve transparency and reporting quality of published research; however, problems with reporting quality persist. This represents a significant barrier to advances in MBI scientific research and translation of findings to practice.

**Objective:**

To update and validate the 2011 reporting guidelines using a rigorous Delphi approach that involved an interdisciplinary group of MBI researchers; and to develop an explanation and elaboration guidance statement to support dissemination and usage.

**Methods:**

We followed the methodological framework for developing reporting guidelines recommended by the EQUATOR Network and guidance recommendations for developing health research reporting guidelines. Our three-stage process included: (1) an initial field scan, (2) a consensus process using Delphi surveys (two rounds) and Expert Panel meetings, and (3) development and dissemination of an explanation and elaboration document.

**Results:**

First-Round survey findings revealed that the original checklist items were capturing content that investigators deemed essential to MBI reporting; however, it also revealed problems with item wording and terminology. Subsequent Expert Panel meetings and the Second-Round survey centered on reaching consensus for item language. The revised RG-MBI checklist has a total of 12-items that pertain to eight different components of MBI interventions including name, theory/scientific rationale, content, interventionist, individual/group, setting, delivery schedule, and treatment fidelity.

**Conclusion:**

We recommend that authors, journal editors, and reviewers use the RG-MBI guidelines, in conjunction with methods-based guidelines (e.g., CONSORT) to accelerate and improve the scientific rigor of MBI research.

## Introduction

Detailed intervention reporting is essential to interpretation, replication, and eventual translation of music-based interventions (MBIs) into practice. Persistent problems with the reporting quality of MBIs represent a significant barrier to advances in scientific research and translation of findings to clinical practice and community settings (Robb et al., 2018; Golden et al., 2021; Chen et al., 2022; Edwards et al., 2023). Interest in the quality of published research reports emerged in the 1980s due to growing awareness about deficiencies in reports of clinical trials at the time (Altman, 1994; Matthews and Rothwell, 2018). For example, several studies at this time found that an increasing number of randomized controlled trials (RCTs) had missing or inaccurate information, such as whether the assessment of outcomes was masked, a primary endpoint specified, or how sample size was determined (Pocock et al., 1987; Matthews and Rothwell, 2018; Sauerbrei et al., 2021). As a result, the use of reporting guidelines was recommended.

Reporting guidelines are a simple, structured tool for health researchers to use while writing manuscripts, which provides a minimum list of information needed to ensure a published manuscript can be understood by a reader, replicated by a researcher, used to inform clinical decisions, and included in systematic reviews (Equator Network, 2024a). The Consolidated Standards for Reporting Trials (CONSORT) and Transparent Reporting of Evaluations with Non-randomized Designs (TREND) guidelines were developed to improve the quality and transparency of published research (Des Jarlais et al., 2004; Schulz et al., 2010). Subsequent publications centered on complexities related to the reporting of behavioral and non-pharmacological interventions, noting that CONSORT and TREND, which have only one item dedicated to intervention reporting, were inadequate (Dijkers et al., 2002; Perera et al., 2007; Boutron et al., 2008a,b). This led to the development of supplemental guidelines specific to intervention reporting, including elaborated CONSORT guidelines for reporting non-pharmacological interventions (Boutron et al., 2008a,b) and the Template for Intervention Description and Replication (TIDieR) checklist (Hoffmann et al., 2014).

Music-based interventions are especially difficult to fully describe, due to the complexity of music stimuli (e.g., rhythm, pitch, tempo, harmonic structure, and timbre), variety of music experiences (e.g., active music making, and music listening) and other factors unique to MBIs. To determine whether intervention reporting guidelines were necessary, Robb and Carpenter (2009) examined how authors described music interventions and found significant gaps in reporting that hinder cross-study comparisons, generalization, and integration of findings into practice. Subsequently, Robb et al. (2011) developed *Reporting Guidelines for Music-Based Interventions* (RG-MBI), which specified components of music interventions that publishing authors were encouraged to report and discuss (Robb et al., 2011). The checklist included 11 items organized across seven component areas including intervention content (five items), theory, delivery schedule, interventionist, treatment fidelity, setting, and unit of delivery (one item each).

The 2011 RG-MBIs are available through the EQUATOR Network (Equator Network, 2024b) and have been referenced by authors in more than 430 publications. However, recent reviews reveal sustained problems with reporting quality (Wang et al., 2018, 2021; Gao et al., 2019; Yangoz and Ozer, 2019, 2022; de Witte et al., 2020, 2022; Duzgun and Ozer, 2020; Moreno-Morales et al., 2020; Bradt et al., 2021; Yang et al., 2021; Jespersen et al., 2022; Monsalve-Duarte et al., 2022; Nguyen et al., 2022). In their 2018 review of MBI reporting quality, Robb et al. (2018) found overall reporting quality was poor with fewer than 50% of authors reporting information for four of the seven checklist components (theory, interventionist qualifications, treatment fidelity, setting). Reporting of intervention content was also poor; again, fewer than 50% of authors reported information about the music used, decibel levels/controls, or materials (Robb et al., 2018).

Sustained problems with reporting quality suggest limited uptake by authors and journal editors of the 2011 music reporting guidelines. This may be due to limited awareness of those guidelines, problems with perceived relevance or clarity of checklist items, and/or the absence of an explanation and elaboration document to provide practical examples across diverse areas of MBI intervention research. Thus, to ensure validity of current checklist items and improve uptake of the reporting guidelines, we completed a rigorous process to update the current guidelines and to establish a process by which to disseminate the resulting validated checklist and guidance statements.

## Methods

We followed the methodological framework for developing reporting guidelines recommended by the EQUATOR Network (Equator Network, 2024c) and recommendations for developing health research reporting guidelines (Moher et al., 2010). The lead author convened a nine-member advisory group that included leaders from the National Institutes of Health (NIH) Music and Health initiative, music intervention researchers, and policy advocates (see acknowledgements). The advisory group worked with authors SR and SS to develop the study protocol and registered the RG-MBI update with the EQUATOR network (Equator Network, 2023). Here we report methods and findings from our three-stage process: (1) field scan, (2) consensus process including Delphi survey and Expert Panel, and (3) resulting modified checklist and planned explanation and elaboration (E&E) guidance statement. This study did not meet criteria for Human Subjects Research and was exempt from Institutional Review Board approval.

### Stage 1: field scan

In 2018, based on items specified in the RG-MBI, Robb et al. (2018) examined reporting quality of published music intervention studies. Overall, reporting quality was determined to be poor, and the terminology used to describe interventions was varied and inconsistent. Golden et al. (2021) found similar problems with reporting, and recommended the generation and uptake of reporting guidelines.

Building on these two reviews, and as our first step, authors SR and JM conducted a field scan of systematic reviews of MBIs published between 2018 and 2022. The purpose of the field scan was to examine and elucidate gaps in reporting quality to inform our Delphi survey and processes. Specifically, we examined whether authors of the systematic reviews discussed reporting quality and, if so, whether they identified additional problems not captured in the current guidelines. We identified 33 systematic reviews, 48% (*n* = 16) of which discussed specific problems with reporting quality. Notably, all the identified problems had been captured by the 2011 MBI reporting guidelines, suggesting limited awareness or uptake of those early guidelines. As such, the field scan findings supported the use of the 2011 RG-MBI checklist as the starting point for a subsequent Delphi Survey process; it also indicated the need to engage stakeholders and interdisciplinary experts to improve content, item clarity, and usage of the guidelines (Supplementary Appendix A).

### Stage 2: item revision and consensus (Delphi Survey and Expert Panel)

The purpose of Stage 2 was to invite music intervention researchers to evaluate content of the 2011 MBI checklist; specifically, they were asked to determine the importance of each item, identify gaps in content, identify problems with wording, and to reach consensus regarding recommended changes to the checklist. Our Delphi process, based on methods described by Sinha et al. (2011), included two survey rounds to reach item consensus, with the plan to add additional rounds as needed (Sinha et al., 2011). Following each survey round, an Expert Panel reviewed all survey data and made final consensus decisions concerning checklist items. In this section we provide details about the Expert Panel, survey participants, and methods for reaching consensus.

#### Participants

##### Interdisciplinary expert panel

The Advisory Group worked with lead authors SR and SS to identify expert panelists with varied expertise and who represent different stakeholder groups engaged in the design, conduct, and dissemination of music and health research. Selection criteria were to identify investigators conducting research: (1) along the translational science continuum, (2) across various domains (sociological, psychological, clinical, community health), (3) with varied methodological expertise, and (4) from a variety of disciplinary backgrounds. This eleven-member panel (authors EE, TG, JJ, DB, MB, JB, CG, AH, JI, ML, and SP) included individuals with expertise in the design, conduct, dissemination, and publication of music and behavioral intervention research from the United States, Europe, and Canada. The group included authors of the original MBI reporting guidelines, journal editors, and researchers with expertise in music cognition and neuroscience, music therapy, intervention research, biostatistics, and community music interventions.

##### Survey participants

Individuals invited to participate in the Delphi survey included United States-based and international experts in music and music-based intervention research. The target sample was comprised of Cochrane review authors, NIH MBI Toolkit panelists, journal editors, authors/investigators (including NIH funded Music and Health grant recipients and authors of systematic reviews identified through our initial field scan), and representatives from patient advocacy and arts organizations. Professional backgrounds included behavioral health, neuroscience, nursing, medicine, music therapy, social work, psychology, and public health. The target sample included 106 experts for Round One and 103 experts for Round Two. Accepting the invitation to complete the survey constituted participants’ consent to participate.

#### Round one survey

The survey opened with a brief overview of the survey purpose, defined key terms, provided an estimated time commitment (including number of rounds), and emphasized the importance of completing each round. Each reporting item from the original guidelines (12 items total), was assigned an identification number to facilitate random ordering. Participants were asked to rate the importance of each item on a four-point Likert Scale (1 = item has limited importance and not required for reporting; 2 = item has moderate importance; 3 = item has high importance; 4 = item has very high importance and essential to reporting). For each item, participants could also provide additional comments or edits to improve the reporting criterion. For items that received a rating of “1 = limited importance” or “4 = very high importance,” we asked participants to provide their rationale for selecting that value and to include any references to support their rationale, if possible. The final two survey items asked participants for additional criteria they believed should be reported in published music intervention research (Question 13) and any additional comments they wanted to share about their responses or the survey (Question 14). See Supplementary Appendix B for survey.

##### Round one data collection and sample

To ensure confidentiality, the Indiana University Center for Survey Research (CSR) distributed and managed survey data using a Qualtrics web survey and recruitment via e-mail. Potential participants were sent an email and invitation; non-respondents and respondents who did not answer all 12 of the first 12 questions received up to two e-mail reminders. To bolster responses, the first author personally e-mailed non-respondents to request their response before the third and final CSR reminder. Additionally, a special reminder with a separate survey link for Questions 13 and 14 only was sent to respondents who partially completed the survey but had not made it to these questions. The first-round survey opened November 3, 2022, and closed January 17, 2023.

The Round One survey was sent to 103 experts for completion after removing three who self-identified as ineligible. The final sample for Round One analysis involved 65 respondents (including partial and complete responses) for a response rate of 63%. Median time to complete the survey was 14 min and an IQR of 23.2 min excluding outliers (>70 min). Outliers included 5 respondents with survey times between 107.90 and 341.42 min, and 6 respondents over 1,160 min. We excluded these cases because they represent individuals who filled out the survey but never submitted it or selected submit after some time with it open in their browser.

##### Round one analysis and expert panel meeting consensus

All data from completed surveys were downloaded to an Excel spreadsheet for descriptive analysis. Likert scores were grouped based on the four response categories: Limited importance (1); Moderate Importance (2); High importance (3); Very high importance (4). For each item, we calculated descriptive statistics for each response category (frequency, percent, valid percent, cumulative valid percent). Consensus criteria for retaining an item was defined as ≥80% of survey respondents rating an item as having “High” or “Very High” importance. Items scoring lower than this threshold were reviewed by the Expert Panel to determine inclusion, removal, and/or refinement of the item for the second-round survey. In addition, comments provided in open-response fields for all items, as well as any suggested additional items (Question 13), were downloaded verbatim for analysis. Two independent reviewers (SR and SS) identified common themes, and then discussed independent findings to reach agreement. In advance of the first Expert Panel meeting, panelist received numeric data, common themes, and representative statements for each item, along with a synthesized list of any new items and related comments.

Expert Meeting panelists were charged with discussing and reaching consensus about: (1) item retention/removal based on numeric and narrative survey data, (2) item level revision based on narrative data, and (3) inclusion of any newly identified items. During meetings, a meeting facilitator invited each panelist to share a unique comment or insight, with an option to pass or affirm another’s comment. Once each panelist had the opportunity to comment, the group assessed whether they had reached consensus. Originally, the authors had identified nominal group technique as its planned approach to reach consensus; however, the panel did not require voting or ranking to achieve consensus on each item.

##### Round one survey and expert panel meeting results

Findings from the Round One survey are available in Supplementary Appendix B. Three items did not reach the threshold score for consensus (≥80% of survey respondents rating the item as having “High” or “Very High” importance); these included Q4: Music (78%), Q6: Intervention Materials (64%), and Q11: Setting (75%). Associated comments pointed to the need for revised language (Q4; Q6), with some suggestions that Q11 could be removed and captured in methods-specific checklists. Consensus from the Expert Panel was that current checklist items were adequate, important, and relevant (no items removed or added). However, there was also consensus that wording/language for all checklist items needed revision, and that the revision process should be the focus of the Round Two Survey. To inform revised item language for the second survey, we used discussion notes from the Expert Panel meeting, and gave panelists time after the meeting to submit more detailed edits. Lead authors (SR, SS) then synthesized these recommendations to create revised item language for the second survey.

#### Round two survey

All eligible participants from the first-round survey (*n* = 102; one person removed by request) were invited to complete the second-round survey which provided a side-by-side comparison of checklist items (original vs. revised). For each item (12 items total), participants were asked to indicate one of three options: (1) prefer original checklist wording; (2) prefer revised checklist wording; (3) a suggested edit (with open text box to provide revised wording). See Supplementary Appendix C for survey.

##### Data collection and sample

Invitation and reminder e-mails followed the same structure and frequency as Round One. The survey opened May 31, 2023, and closed July 18, 2023. The final sample for Round Two analysis involved 61 respondents (including partial and complete responses) for a response rate of 60%. Median time to complete the survey was 7 min and an IQR of 5.3 min excluding outliers (> 70 min). Outliers included 9 respondents with survey times between 70.5 and 965.5 min, and 9 respondents over 3,273 min. We excluded these cases because they represent individuals who filled out the survey but never submitted it or selected submit after some time with it open in their browser.

##### Second round analysis

To determine whether there was consensus for original or revised items we calculated frequency, mean, and percent scores for each item. Consensus was defined as items that were selected by ≥80% of survey respondents. In addition, the panel used open-ended comments from survey respondents to determine if an item required further revision. The Expert Panel’s charge was to review items that did not reach consensus using discussion as well as survey respondents’ open-ended comments to inform final changes to item wording, sentence structure, or organization.

##### Results round two survey and expert panel meetings

Findings from the Round Two survey are available in Supplementary Appendix C. Survey respondents preferred revised language for all items; however, three items did not reach the threshold for consensus (≥80% of respondents preferred revised item language): Q2: Person Selecting the Music (63%); Q3: Music (74%); Q9: Treatment Fidelity (52%). For all items, we received suggestions on how we could improve item language. The Expert Panel had two subsequent meetings in which they discussed survey respondent recommendations, terminology, whether to include embedded examples, and the ultimate order of checklist items (including alignment with TIDieR and CONSORT Non-Pharmacological checklists). All Expert Panel decisions were made using our *a priori* consensus threshold of ≥80% agreement.

## Revised reporting guidelines for music-based interventions

The revised Reporting Guidelines for Music-Based Interventions appear in Table 1.

**Table 1:** Reporting Guidelines for Music-based Interventions checklist^a^.

Item number		Item	Location^b^ (page or appendix number)
1		**Brief Name**^c^ Provide the name or phrase that describes the intervention.	
2		**Intervention Theory and/or Scientific Rationale** Provide a rationale for the music and/or music experience(s). Specify how essential features of the music and music experience(s) are expected to influence targeted outcomes.	
3		**Intervention Content** For Items 3a–3e, describe the music intervention with enough detail to support replication. When applicable, describe procedures for tailoring the intervention.	
	3a	**Music Selection** Describe the process for how music was selected including who was involved in music selection.	
	3b	**Music** Specify key details about the music that may be relevant to specified outcomes of interest. Characteristics may include compositional features of the music (such as tempo, harmony, rhythm, pitch, tonality, form, instrumentation),^d^ sound intensity or volume, lyrics, and/or how the music relates to the participants’ cultural identity and heritage. When using published music, provide reference for a sound recording or sheet music.	
	3c	**Music Delivery Method** Provide details about how music was provided to or created with participants (such as live, recorded, computer generated).^d^ Include any details necessary for replication. This might include size of performing group, use of playback equipment, or person controlling volume.	
	3d	**Materials** List all materials necessary for the music experience. Include music and non-music equipment and materials.	
	3e	**Intervention Strategies** Describe the music intervention strategy or strategies being studied (such as music listening, improvisation, song writing, rhythmic auditory stimulation).^d^	
4		**Interventionist** Specify interventionist qualifications, credentials, training, and/or experience. Indicate how many interventionists delivered the music experience.	
5		**Individual or Group Intervention** Specify whether interventions were delivered to individuals or groups of individuals. For group interventions, specify the size of the group.	
6		**Setting** Describe where the intervention was delivered. Include location, privacy level, ambient sound, and/or any other factors that may have affected participants’ experiences.	
7		**Intervention Delivery Schedule** Report number of sessions, session length (for example, 60 min), frequency (for example, 3×/week), time interval between sessions (for example, single day, three consecutive days), and duration (for example, over 4 weeks).^d^ Include practice, experiences, or tasks that are assigned to participants between intervention sessions.	
8		**Treatment Fidelity** Describe strategies and/or measures used to ensure that the music intervention was delivered and received as intended.	

a We recommend using this checklist in conjunction with the Reporting Guidelines for Music-Based Intervention guide (Robb et al., 2025) which contains an explanation and elaboration for each item. The focus of the RG-MBI is on reporting details of the music-based intervention under investigation. Importantly, the checklist was designed to be used in conjunction with methodological checklists such as CONSORT (for randomized controlled trials), SPIRIT for clinical trial protocols, and other study designs (see www.equator-netowrk.org). For example, when reporting findings from a randomized controlled trial, the RGMBI checklist can serve as an extension of Item 5: Interventions on the CONSORT 2010 checklist.

b Use N/A if an item is not applicable for the intervention being described.

c Item 1 is taken from the TIDieR checklist. Following RGMBI item validation, we ordered RGMBI Items 2–8 to coincide with the order of TIDieR items based on content.

d Parenthetical details are examples only; they are not intended to be exhaustive. See the RGMBI explanation and elaboration document for additional examples (Robb et al., 2025).

## Discussion

The 2011 Reporting Guidelines for Music-Based Interventions were developed to improve transparency and reporting quality of published research. Despite an increased number of publications citing the guidelines, recent reviews indicate persistent problems with reporting quality. Incomplete and inconsistent reporting of MBIs impedes cross-study comparisons, interpretation, replication, and application of findings to clinical practice and community-based programming.

To improve uptake of the RG-MBIs by a larger and more diverse group of MBI researchers, we convened a team of experts from diverse disciplines to engage in a rigorous Delphi study process. This process revealed that the original checklist items were indeed capturing content that investigators deemed essential to MBI reporting; however, it also identified important problems with existing items that may have been affecting its uptake and effective usage. In particular, findings indicted changes in wording and terminology that would allow checklist items to be inclusive of a wide range of music experiences (e.g., music as a sound stimulus and creating music/musicking) and approaches (e.g., social, psychological, physical, neurological, and biological). The illumination of these issues resulted in robust discussion among Expert Panelists and several rounds of revisions to item language in the guidelines. By engaging an international and diverse group of experts to revise item language, our expectation is that the revised checklist will be clearer, easier to apply, and of greater relevance for a diverse group of MBI investigators.

To further facilitate usage, items were re-ordered to align with the TIDieR checklist including the addition of item one from the TIDieR checklist (Hoffmann et al., 2014). Expert panel members also co-authored an Explanation and Elaboration (E&E) guidance document to companion the revised RG-MBI (Robb et al., 2025). This document includes a rationale for each item, concrete instructions for optimally reporting each item, and annotated examples from published manuscripts. Our expectation is that the revised RG-MBI will be of greater utility to investigators across a wider range of disciplines and that the E&E document will support greater adoption of the RG-MBI by authors and journal editors.

A primary limitation of this validation study was limited representation of investigators and stakeholders from countries outside the United States. Reliance on systematic reviews, Cochrane Reviews, journal editors, and US-based research initiatives to generate our survey sample did not ensure representation of music and health researchers, clinicians, and advocates at a global level. Second, we did not obtain information about survey respondents’ professional background and country, limiting our ability to assess representation. Finally, we did not conduct a formal study to investigate researchers’ awareness of the 2011 RG-MBIs to gain further insight into specific barriers to adoption.

We recommend that authors, journal editors, and reviewers use the RG-MBI guidelines, in conjunction with methods-based guidelines like CONSORT and TREND, to accelerate and improve the scientific rigor of MBI research. We also recommend a review of MBI reporting quality in 5 years to evaluate the impact of the revised guidelines and subsequent international studies centered on RG-MBI utility, along with barriers and facilitators to their adoption.

## Data Availability

The original contributions presented in the study are included in the article/Supplementary material, further inquiries can be directed to the corresponding author.
