# Correction: Reporting Guidelines for Music-based Interventions: an update and validation study

**DOI:** 10.3389/fpsyg.2025.1663581

**Published:** 2025-07-30

**Authors:** Frontiers Production Office

**Affiliations:** Frontiers Media SA, Lausanne, Switzerland

**Keywords:** reporting guidelines, music, music therapy, intervention, reporting quality

Due to an error during the finalisation of the article, the article title and a heading in the Methods section were published incorrectly.

The article title should state “Interventions,” not “Intervention.” The full corrected title is shown below:

“Correction: Reporting Guidelines for Music-based Interventions: an update and validation study”

The incorrect heading in section “Methods”, “Stage 2: item revision and consensus (Delphi Survey and Expert Panel)”, “Round one survey”, was published as “Round two survey and expert panel meeting results”. The corrected heading is shown below:

“Round one data collection and sample”

The original version of this article has been updated.

