# Supplementary material for: Reporting Guidelines for Music-based Interventions: an update and validation study
Source: Front Psychol. 2025 Jun 2;16:1551920. doi: 10.3389/fpsyg.2025.1551920 (PMC12171218; doi:10.3389/fpsyg.2025.1551920)
Supplement: Supplementary file 1 [file Supplementary_file_1.docx]

**Supplemental Material**

**Appendix A: Field Scan**

**Field Scan Flowchart.**

**Search Terms:**

- “Review” in Title and [“music therapy” or “music intervention”]
- 2018-2022
- Review or Systematic Review publication type

**Databases Searched:**

- PubMed
- CINAHL
- PsycINFO
- Cochrane
- JBI EBP

**Identified**: 104

**Excluded**: 71

- 17 duplicates
- 2 due to language
- 2 corrigenda
- 50 non-meta-analyses

**Included**: 33

**Field Scan Table.** Music-Based Intervention Review Articles (2018-2022). Table indicates whether authors discussed areas for improved reporting and whether the authors referenced the 2011 *Reporting Guidelines for Music-Based Interventions*.

| **Authors** | **Authors Discussed Areas for Improved Reporting**  ***Referenced the Reporting Guidelines**** | **Authors Discussed Areas for Improved Reporting**  ***No Reference to Reporting Guidelines**** |
| --- | --- | --- |
| Chen et al^1^ | -- | -- |
| Chuang et al^2^ | -- | -- |
| de Witte et al^3^ | Intervention content; treatment fidelity; | -- |
| de Witte et al^4^ | Intervention content; Intervention delivery schedule; Interventionist; Setting | -- |
| Düzgün & Özer^5^ | Intervention content; Intervention delivery schedule | Intervention content; Intervention delivery schedule |
| Fusar-Poli et al^6^ | -- | Interventionist qualifications |
| Gao et al^7^ | Person selecting music | Person selecting music |
| He et al^8^ | -- | -- |
| Kakar et al^9^ | -- | Intervention content; Intervention delivery schedule |
| Kakar et al^10^ | -- | Intervention content; Intervention delivery schedule |
| Köhler et al^11^ | -- | Intervention delivery schedule; Recipient musical background |
| Li et al^12^ | -- | Interventionist qualifications; Intervention setting |
| Li et al^13^ | -- | Intervention delivery schedule |
| Lieber et al^14^ | -- | Interventionist qualifications |
| Liu et al^15^ | -- | -- |
| Mahmoud et al^16^ | -- | -- |
| Mishra et al^17^ | -- | -- |
| Monsalve-Duarte et al^18^ | Intervention content; Music | -- |
| Moreno-Morales et al^19^ | Intervention content; person selecting the music | Intervention theory |
| Nguyen et al^20^ | Intervention theory | Intervention theory |
| Patiyal et al^21^ | -- | -- |
| Santiváñez-Acosta et al^22^ | -- | -- |
| Ting et al^23^ | -- | Music; Music delivery method |
| Tsoi et al^24^ | -- | Intervention delivery schedule |
| Wang et al^25^ | Intervention content; Intervention delivery schedule; Interventionist qualifications | Intervention theory |
| Wang et al^26^ | Interventionist qualifications | Intervention theory |
| Wang et al^27^ | -- | -- |
| Wu et al^28^ | -- | Intervention theory |
| Wu et al^29^ | -- | -- |
| Yang et al^30^ | Intervention content | Music |
| Yangöz & Özer^31^ | Intervention content | Intervention content |
| Yangöz & Özer^32^ | Music | -- |
| Yue et al^33^ | -- | Intervention delivery schedule; Interventionist qualifications |

*Robb SL, Carpenter JS, Burns DS. Reporting Guidelines for Music-based Interventions. *Journal of Health Psychology*. 2011;16(2):342-352. doi:[10.1177/1359105310374781](https://doi.org/10.1177/1359105310374781)

**References**

1. Chen C-T, Tung H-H, Fang C-J, et al. Effect of music therapy on improving sleep quality in older adults: a systematic review and meta-analysis. *J Am Geriatr Soc* 2021;69(7):1925-1932. doi:10.1111/jgs.17149

2. Chuang C-H, Chen P-C, Lee CS, Chen C-H, Tu Y-K, Wu S-C. Music intervention for pain and anxiety management of the primiparous women during labour: a systematic review and meta-analysis. *J Adv Nurs*. 2019;75(4):723-733. doi:10.1111/jan.13871

3. de Witte M, Pinho AdS, Stams G-J, Moonen X, Bos AER, van Hooren S. Music therapy for stress reduction: a systematic review and meta-analysis. *Health Psychol Rev*. 2022/01/02 2022;16(1):134-159. doi:10.1080/17437199.2020.1846580

4. de Witte M, Spruit A, van Hooren S, Moonen X, Stams G-J. Effects of music interventions on stress-related outcomes: a systematic review and two meta-analyses. *Health Psychol Rev*. 2020/04/02 2020;14(2):294-324. doi:10.1080/17437199.2019.1627897

5. Düzgün MV, Özer Z. The effects of music ıntervention on breast milk production in breastfeeding mothers: a systematic review and meta-analysis of randomized controlled trials. *J Adv Nurs*. 2020;76(12):3307-3316. doi:10.1111/jan.14589

6. Fusar-Poli L, Bieleninik Ł, Brondino N, Chen X-J, Gold C. The effect of music therapy on cognitive functions in patients with dementia: a systematic review and meta-analysis. *Aging Ment Health*. 2018;22(9):1103-1112. doi:10.1080/13607863.2017.1348474

7. Gao Y, Wei Y, Yang W, et al. The effectiveness of music therapy for terminally ill patients: a meta-analysis and systematic review. *J Pain Symptom Manage*. 2019;57(2):319-329. doi:10.1016/j.jpainsymman.2018.10.504

8. He H, Huang J, Zhao X, Li Z. The effect of prenatal music therapy on fetal and neonatal status: a systematic review and meta-analysis. *Complement Ther Med*. 2021;60:102756. doi:10.1016/j.ctim.2021.102756

9. Kakar E, Billar RJ, van Rosmalen J, Klimek M, Takkenberg JJM, Jeekel J. Music intervention to relieve anxiety and pain in adults undergoing cardiac surgery: a systematic review and meta-analysis. *Open Heart*. 2021;8(1):e001474. doi:10.1136/openhrt-2020-001474

10. Kakar E, Venema E, Jeekel J, Klimek M, van der Jagt M. Music intervention for sleep quality in critically ill and surgical patients: a meta-analysis. *BMJ Open*. 2021;11(5):e042510. doi:10.1136/bmjopen-2020-042510

11. Köhler F, Martin Z-S, Hertrampf R-S, et al. Music therapy in the psychosocial treatment of adult cancer patients: a systematic review and meta-analysis. *Front Psychol*. 2020;11doi:10.3389/fpsyg.2020.00651

12. Li H-C, Wang H-H, Lu C-Y, Chen T-B, Lin Y-H, Lee I. The effect of music therapy on reducing depression in people with dementia: a systematic review and meta-analysis. *Geriatr Nurs*. 2019;40(5):510-516. doi:10.1016/j.gerinurse.2019.03.017

13. Li X, Li C, Hu N, Wang T. Music interventions for disorders of consciousness: a systematic review and meta-analysis. *J Neurosci Nurs*. 2020;52(4):146-151. doi:10.1097/jnn.0000000000000511

14. Lieber AC, Bose J, Zhang X, et al. Effects of music therapy on anxiety and physiologic parameters in angiography: a systematic review and meta-analysis. *J Neurointerv Surg*. 2019;11(4):416-423. doi:10.1136/neurintsurg-2018-014313

15. Liu Q, Li W, Yin Y, et al. The effect of music therapy on language recovery in patients with aphasia after stroke: a systematic review and meta-analysis. *Neurol Sci*. 2022;43(2):863-872. doi:10.1007/s10072-021-05743-9

16. Mahmoud MY, Labib K, Sileem SA, et al. The impact of music therapy on anxiety and pregnancy rate among infertile women undergoing assisted reproductive technologies: a systematic review and meta-analysis. *J Psychosom Obstet Gynaecol*. 2022;43(2):205-213. doi:10.1080/0167482X.2021.1977277

17. Mishra R, Florez-Perdomo WA, Shrivatava A, et al. Role of music therapy in traumatic brain injury: a systematic review and meta-analysis. *World Neurosurg*. 2021;146:197-204. doi:10.1016/j.wneu.2020.10.130

18. Monsalve-Duarte S, Betancourt-Zapata W, Suarez-Cañon N, et al. Music therapy and music medicine interventions with adult burn patients: a systematic review and meta-analysis. *Burns*. 2022;48(3):510-521. doi:10.1016/j.burns.2021.11.002

19. Moreno-Morales C, Calero R, Moreno-Morales P, Pintado C. Music Therapy in the Treatment of Dementia: A Systematic Review and Meta-Analysis. Systematic Review. *Front Med*. 2020;7. doi:10.3389/fmed.2020.00160

20. Nguyen KT, Xiao J, Chan DNS, Zhang M, Chan CWH. Effects of music intervention on anxiety, depression, and quality of life of cancer patients receiving chemotherapy: a systematic review and meta-analysis. *Support Care Cancer*. 2022;30(7):5615-5626. doi:10.1007/s00520-022-06881-2

21. Patiyal N, Kalyani V, Mishra R, et al. Effect of music therapy on pain, anxiety, and use of opioids among patients underwent orthopedic surgery: a systematic review and meta-analysis. *Cureus*. 2021;13(9):e18377. doi:10.7759/cureus.18377

22. Santiváñez-Acosta R, Tapia-López EdlN, Santero M. Music therapy in pain and anxiety management during labor: a systematic review and meta-analysis. *Medicina*. 2020;56(10):526. doi:10.3390/medicina56100526

23. Ting B, Tsai C-L, Hsu W-T, et al. Music intervention for pain control in the pediatric population: a systematic review and meta-analysis. *J Clin Med*. 2022;11(4):991. doi:10.3390/jcm11040991

24. Tsoi KKF, Chan JYC, Ng Y-M, Lee MMY, Kwok TCY, Wong SYS. Receptive music therapy is more effective than interactive music therapy to relieve behavioral and psychological symptoms of dementia: a systematic review and meta-analysis. *J Am Med Dir Assoc*. 2018;19(7):568-576.e3. doi:10.1016/j.jamda.2017.12.009

25. Wang C, Li G, Zheng L, et al. Effects of music intervention on sleep quality of older adults: a systematic review and meta-analysis. *Complement Ther Med*. 2021;59:102719. doi:10.1016/j.ctim.2021.102719

26. Wang X, Zhang Y, Fan Y, Tan X-S, Lei X. Effects of music intervention on the physical and mental status of patients with breast cancer: a systematic review and meta-analysis. *Breast Care*. 2018;13(3):183-190. doi:10.1159/000487073

27. Wang Y, Wei J, Guan X, et al. Music intervention in pain relief of cardiovascular patients in cardiac procedures: a systematic review and meta-analysis. *Pain Med*. 2020;21(11):3055-3065. doi:10.1093/pm/pnaa148

28. Wu T-J, Chen K-H, Chiu W-K, et al. Optimal timing and effect of music therapy in patients with burn injuries: systematic review and meta-analysis of randomized controlled trials. *Burns*. 2022;48(5):1069-1078. doi:10.1016/j.burns.2021.07.016

29. Wu XL, Ji B, Yao SD, Wang LL, Jiang ZY. Effect of music intervention during hemodialysis: a comprehensive meta-analysis. *Eur Rev Med Pharmacol Sci*. 2021;25(10):3822-3834. doi:10.26355/eurrev_202105_25950

30. Yang T, Wang S, Wang R, et al. Effectiveness of five-element music therapy in cancer patients: a systematic review and meta-analysis. *Complement Ther Clin Pract*. 2021;44:101416. doi:10.1016/j.ctcp.2021.101416

31. Yangöz ŞT, Özer Z. The effect of music intervention on patients with cancer-related pain: a systematic review and meta-analysis of randomized controlled trials. *J Adv Nurs*. 2019;75(12):3362-3373. doi:https://doi.org/10.1111/jan.14184

32. Yangöz ŞT, Özer Z. Effects of music intervention on physical and psychological problems in adults receiving haemodialysis treatment: A systematic review and meta-analysis. *Journal of Clinical Nursing*. 2022;31(23-24):3305-3326. doi:10.1111/jocn.16199

33. Yue W, Han X, Luo J, Zeng Z, Yang M. Effect of music therapy on preterm infants in neonatal intensive care unit: Systematic review and meta-analysis of randomized controlled trials. *Journal of Advanced Nursing*. 2021;77(2):635-652. doi:10.1111/jan.14630
