# Supplementary material for: Reporting Guidelines for Music-based Interventions: an update and validation study
Source: Front Psychol. 2025 Jun 2;16:1551920. doi: 10.3389/fpsyg.2025.1551920 (PMC12171218; doi:10.3389/fpsyg.2025.1551920)
Supplement: Supplementary file 2 [file Supplementary_file_2.docx]

**Supplemental Material**

**Appendix B: Round One Survey & Round One Survey Results**

Reporting Guidelines for Music-based Interventions Questionnaire Fall 2022

Instructions The *Reporting Guidelines* provide a minimum list of information needed to ensure a published manuscript can be understood by the reader, replicated by a researcher, used to inform clinical decisions, and included in systematic reviews. These *Guidelines* focus on the description of the music intervention and are intended to be used in conjunction with methods-specific reporting guidelines (e.g., CONSORT, TREND).

**Please think about the following *Music-based Intervention Reporting Criteria* and rate the importance of each criterion on a scale of 1 to 4. Provide comments, as necessary, to improve the criterion definition.**
  
 • 1= criterion has limited importance; not required for reporting,
 • 2= criterion has moderate importance,
 • 3= criterion has high importance,
 • 4= criterion has very high importance; essential for reporting

PAGE BREAK

*NOTE: Question blocks 1-12, which includes open-text Questions 1a-12a, were randomly presented to respondents.*

Q1 Please rate the importance of the **Intervention Theory** criterion on a scale of 1 to 4. 

**Intervention Theory.** Provide a rationale for the music selected; specify how qualities and delivery of the music are expected to impact targeted outcomes.

- **Limited importance 1** (1)
- **Moderate importance 2** (2)
- **High importance 3** (3)
- **Very high importance 4** (4)

Q1a Please provide additional comments or edits that you feel would improve this reporting criterion**.** *If none, please leave blank.*

________________________________________________________________

________________________________________________________________

________________________________________________________________

PAGE BREAK

Q2 Please rate the importance of the **Intervention Content** criterion on a scale of 1 to 4. 

**Intervention Content.** Provide precise details of the music intervention and, when applicable, descriptions of procedures for tailoring interventions to individual participants.

- **Limited importance 1** (1)
- **Moderate importance 2** (2)
- **High importance 3** (3)
- **Very high importance 4** (4)

Q2a Please provide additional comments or edits that you feel would improve this reporting criterion. *If none, please leave blank.*

________________________________________________________________

________________________________________________________________

________________________________________________________________

PAGE BREAK

Q3 Please rate the importance of the **Intervention Content – Person Selecting the Music**criterion on a scale of 1 to 4. 

**Intervention Content – Person Selecting the Music.** Specify who selected the music: (1) pre-selected by investigators, (2) participant selected from limited set, (3) participant selected from own collections, or (4) tailored based on patient assessment.

- **Limited importance 1** (1)
- **Moderate importance 2** (2)
- **High importance 3** (3)
- **Very high importance 4** (4)

Q3a Please provide additional comments or edits that you feel would improve this reporting criterion. *If none, please leave blank.*

________________________________________________________________

________________________________________________________________

________________________________________________________________

PAGE BREAK

Q4 Please rate the importance of the **Intervention Content – Music**criterion on a scale of 1 to 4. 

**Intervention Content – Music.** When using published music, provide reference for sheet music or sound recording. When using improvised or original music, describe the music’s overall structure (i.e., form, elements, instruments, etc.).

- **Limited importance 1** (1)
- **Moderate importance 2** (2)
- **High importance 3** (3)
- **Very high importance 4** (4)

Q4a Please provide additional comments or edits that you feel would improve this reporting criterion. *If none, please leave blank.*

________________________________________________________________

________________________________________________________________

________________________________________________________________

PAGE BREAK

Q5 Please rate the importance of the **Intervention Content – Music Delivery Method (Live or Recorded)**criterion on a scale of 1 to 4. 

**Intervention Content – Music Delivery Method (Live or Recorded)**. When using live music, specify who delivered the music and the size of the performance group (e.g., interventionist only, interventionist and participant). When using recorded music, specify placement of playback equipment and the use of headphones vs. speakers. Specify who determined/controlled volume (e.g., interventionist, participant). Specify decibel level of music delivered and/or use of volume controls to limit decibels.

- **Limited importance 1** (1)
- **Moderate importance 2** (2)
- **High importance 3** (3)
- **Very high importance 4** (4)

Q5a Please provide additional comments or edits that you feel would improve this reporting criterion. *If none, please leave blank.*

________________________________________________________________

________________________________________________________________

________________________________________________________________

PAGE BREAK

Q6 Please rate the importance of the **Intervention Content – Intervention Materials**criterion on a scale of 1 to 4. 

**Intervention Content – Intervention Materials**. Specify music and non-music materials.

- **Limited importance 1** (1)
- **Moderate importance 2** (2)
- **High importance 3** (3)
- **Very high importance 4** (4)

Q6a Please provide additional comments or edits that you feel would improve this reporting criterion. *If none, please leave blank.*

________________________________________________________________

________________________________________________________________

________________________________________________________________

PAGE BREAK

Q7 Please rate the importance of the **Intervention Content – Intervention Strategies**criterion on a scale of 1 to 4. 

**Intervention Content – Intervention Strategies**. Describe music-based intervention strategies under investigation (e.g., music listening, songwriting, improvisation, lyric analysis, rhythmic auditory stimulation, etc.).

- **Limited importance 1** (1)
- **Moderate importance 2** (2)
- **High importance 3** (3)
- **Very high importance 4** (4)

Q7a Please provide additional comments or edits that you feel would improve this reporting criterion. I*f none, please leave blank.*

________________________________________________________________

________________________________________________________________

________________________________________________________________

PAGE BREAK

Q8 Please rate the importance of the **Intervention Delivery Schedule**criterion on a scale of 1 to 4. 

**Intervention Delivery Schedule.** Report number of sessions, session duration, and session frequency including homework.

- **Limited importance 1** (1)
- **Moderate importance 2** (2)
- **High importance 3** (3)
- **Very high importance 4** (4)

Q8a Please provide additional comments or edits that you feel would improve this reporting criterion. *If none, please leave blank.*

________________________________________________________________

________________________________________________________________

________________________________________________________________

PAGE BREAK

Q9 Please rate the importance of the **Interventionist**criterion on a scale of 1 to 4.

**Interventionist.**Specify interventionist qualifications and/or credentials. Specify how many interventionists delivered study conditions.

- **Limited importance 1** (1)
- **Moderate importance 2** (2)
- **High importance 3** (3)
- **Very high importance 4** (4)

Q9a Please provide additional comments or edits that you feel would improve this reporting criterion. *If none, please leave blank.*

________________________________________________________________

________________________________________________________________

________________________________________________________________

PAGE BREAK

Q10 Please rate the importance of the **Treatment Fidelity** criterion on a scale of 1 to 4. 

**Treatment Fidelity.** Describe strategies used to ensure that treatment and/or control conditions were delivered as intended (e.g., interventionist training, manualized protocols, intervention monitoring).

- **Limited importance 1** (1)
- **Moderate importance 2** (2)
- **High importance 3** (3)
- **Very high importance 4** (4)

Q10a Please provide additional comments or edits that you feel would improve this reporting criterion. *If none, please leave blank.*

________________________________________________________________

________________________________________________________________

________________________________________________________________

PAGE BREAK

Q11 Please rate the importance of the **Setting** criterion on a scale of 1 to 4. 

**Setting.** Describe where the intervention was delivered; include location, privacy level, and ambient sound.

- **Limited importance 1** (1)
- **Moderate importance 2** (2)
- **High importance 3** (3)
- **Very high importance 4** (4)

Q11a Please provide additional comments or edits that you feel would improve this reporting criterion. *If none, please leave blank.*

________________________________________________________________

________________________________________________________________

________________________________________________________________

PAGE BREAK

Q12 Please rate the importance of the **Unit of Delivery** criterion on a scale of 1 to 4. 

**Unit of Delivery**. Specify whether interventions were delivered to individuals or groups of individuals, including the size of the group.

- **Limited importance 1** (1)
- **Moderate importance 2** (2)
- **High importance 3** (3)
- **Very high importance 4** (4)

Q12a Please provide additional comments or edits that you feel would improve this reporting criterion**.** *If none, please leave blank.*

________________________________________________________________

________________________________________________________________

________________________________________________________________

PAGE BREAK

Display This Text:

If any response to Q1 - Q12 = Limited importance 1

Next, we ask that you provide a rationale for the criteria that you rated as having **"1 = Limited importance"** on the importance scale. Although not required, if you have a reference to support your rationale, please include that as well.

Display This Question:

If Q1 = Limited importance 1

Q1b You selected **"1 = Limited importance"** to:

***Intervention Theory.*** *Provide a rationale for the music selected; specify how qualities and delivery of the music are expected to impact targeted outcomes.*

Please provide your rationale for selecting this value. Include a reference to support your rationale, if possible.

________________________________________________________________

________________________________________________________________

________________________________________________________________

Display This Question:

If Q2 = Limited importance 1

Q2b You selected **"1 = Limited importance"** to:

***Intervention Content.****Provide precise details of the music intervention and, when applicable, descriptions of procedures for tailoring interventions to individual participants.*

Please provide your rationale for selecting this value. Include a reference to support your rationale, if possible.

____________________________________________________________

________________________________________________________________

________________________________________________________________

Display This Question:

If Q3 = Limited importance 1

Q3b You selected **"1 = Limited importance"**to:

***Intervention Content – Person Selecting the Music****. Specify who selected the music: (1) pre-selected by investigators, (2) participant selected from limited set, (3) participant selected from own collections, or (4) tailored based on patient assessment.*

Please provide your rationale for selecting this value. Include a reference to support your rationale, if possible.

_____________________________________________________________

________________________________________________________________

________________________________________________________________

Display This Question:

If Q4 = Limited importance 1

Q4b You selected **"1 = Limited importance"**to:

***Intervention Content – Music.*** *When using published music, provide reference for sheet music or sound recording. When using improvised or original music, describe the music’s overall structure (i.e., form, elements, instruments, etc.).*

Please provide your rationale for selecting this value. Include a reference to support your rationale, if possible.

________________________________________________________________

________________________________________________________________

________________________________________________________________

Display This Question:

If Q5 = Limited importance 1

Q5b You selected **"1 = Limited importance"**to:

***Intervention Content – Music Delivery Method (Live or Recorded).*** *When using live music, specify who delivered the music and the size of the performance group (e.g., interventionist only, interventionist and participant). When using recorded music, specify placement of playback equipment and the use of headphones vs. speakers. Specify who determined/controlled volume (e.g., interventionist, participant). Specify decibel level of music delivered and/or use of volume controls to limit decibels.*

Please provide your rationale for selecting this value. Include a reference to support your rationale, if possible.

________________________________________________________________

________________________________________________________________

________________________________________________________________

Display This Question:

If Q6 = Limited importance 1

Q6b You selected **"1 = Limited importance"**to:

***Intervention Content – Intervention Materials.*** *Specify music and non-music materials.*

Please provide your rationale for selecting this value. Include a reference to support your rationale, if possible.

________________________________________________________________

________________________________________________________________

________________________________________________________________

Display This Question:

If Q7 = Limited importance 1

Q7b You selected **"1 = Limited importance"**to:

***Intervention Content – Intervention Strategies.*** *Describe music-based intervention strategies under investigation (e.g., music listening, songwriting, improvisation, lyric analysis, rhythmic auditory stimulation, etc.).*

Please provide your rationale for selecting this value. Include a reference to support your rationale, if possible.

________________________________________________________________

________________________________________________________________

________________________________________________________________

Display This Question:

If Q8 = Limited importance 1

Q8b You selected **"1 = Limited importance"**to:

***Intervention Delivery Schedule.*** *Report number of sessions, session duration, and session frequency including homework.*

Please provide your rationale for selecting this value. Include a reference to support your rationale, if possible.
   ________________________________________________________________

________________________________________________________________

________________________________________________________________

Display This Question:

If Q9 = Limited importance 1

Q9b You selected **"1 = Limited importance"**to:

***Interventionist.*** *Specify interventionist qualifications and/or credentials. Specify how many interventionists delivered study conditions.*
Please provide your rationale for selecting this value. Include a reference to support your rationale, if possible.

_______________________________________________________________

________________________________________________________________

________________________________________________________________

Display This Question:

If Q10 = Limited importance 1

Q10b You selected **"1 = Limited importance"**to:

***Treatment Fidelity.*** *Describe strategies used to ensure that treatment and/or control conditions were delivered as intended (e.g., interventionist training, manualized protocols, intervention monitoring).*
Please provide your rationale for selecting this value. Include a reference to support your rationale, if possible.

________________________________________________________________

________________________________________________________________

________________________________________________________________

Display This Question:

If Q11 = Limited importance 1

Q11b You selected **"1 = Limited importance"**to:

***Setting.*** *Describe where the intervention was delivered; include location, privacy level, and ambient sound.*

 Please provide your rationale for selecting this value. Include a reference to support your rationale, if possible.

________________________________________________________________

________________________________________________________________

________________________________________________________________

Display This Question:

If Q12 = Limited importance 1

Q12b You selected **"1 = Limited importance"**to:

***Unit of Delivery.*** *Specify whether interventions were delivered to individuals or groups of individuals, including the size of the group.*

Please provide your rationale for selecting this value. Include a reference to support your rationale, if possible.

________________________________________________________________

________________________________________________________________

________________________________________________________________

Display This Text:

If any response to Q1 – Q12 = Very high importance 4

Next, we ask that you provide a rationale for criteria that you rated as having **"4 = Very high importance"** on the importance scale. Although not required, if you have a reference to support your rationale, please include that as well.

Display This Question:

If Q1 = Very high importance 4

Q1c You selected **"4 = Very high importance"**to:

***Intervention Theory.*** *Provide a rationale for the music selected; specify how qualities and delivery of the music are expected to impact targeted outcomes.*

Please provide your rationale for selecting this value. Include a reference to support your rationale, if possible.

________________________________________________________________

________________________________________________________________

________________________________________________________________

Display This Question:

If Q2 = Very high importance 4

Q2c You selected **"4 = Very high importance"**to:

***Intervention Content.*** *Provide precise details of the music intervention and, when applicable, descriptions of procedures for tailoring interventions to individual participants.*
Please provide your rationale for selecting this value. Include a reference to support your rationale, if possible.

________________________________________________________________

________________________________________________________________

Display This Question:

If Q3 = Very high importance 4

Q3c You selected **"4 = Very high importance"**to:

***Intervention Content – Person Selecting the Music.*** *Specify who selected the music: (1) pre-selected by investigators, (2) participant selected from limited set, (3) participant selected from own collections, or (4) tailored based on patient assessment.*

Please provide your rationale for selecting this value. Include a reference to support your rationale, if possible.

_______________________________________________________________

________________________________________________________________

________________________________________________________________

Display This Question:

If Q4 = Very high importance 4

Q4c You selected **"4 = Very high importance"**to:

***Intervention Content – Music.*** *When using published music, provide reference for sheet music or sound recording. When using improvised or original music, describe the music’s overall structure (i.e., form, elements, instruments, etc.).*

Please provide your rationale for selecting this value. Include a reference to support your rationale, if possible.

_______________________________________________________________

________________________________________________________________

________________________________________________________________

Display This Question:

If Q5 = Very high importance 4

Q5c You selected **"4 = Very high importance"**to:

***Intervention Content – Music Delivery Method (Live or Recorded).*** *When using live music, specify who delivered the music and the size of the performance group (e.g., interventionist only, interventionist and participant). When using recorded music, specify placement of playback equipment and the use of headphones vs. speakers. Specify who determined/controlled volume (e.g., interventionist, participant). Specify decibel level of music delivered and/or use of volume controls to limit decibels.*

Please provide your rationale for selecting this value. Include a reference to support your rationale, if possible.

_____________________________________________________________

_____________________________________________________________

_____________________________________________________________

Display This Question:

If Q6 = Very high importance 4

Q6c You selected **"4 = Very high importance"**to:

***Intervention Content – Intervention Materials****. Specify music and non-music materials.*

Please provide your rationale for selecting this value. Include a reference to support your rationale, if possible.

________________________________________________________________

________________________________________________________________

________________________________________________________________

Display This Question:

If Q7 = Very high importance 4

Q7c You selected **"4 = Very high importance"**to:

***Intervention Content – Intervention Strategies.*** *Describe music-based intervention strategies under investigation (e.g., music listening, songwriting, improvisation, lyric analysis, rhythmic auditory stimulation, etc.).*

Please provide your rationale for selecting this value. Include a reference to support your rationale, if possible.

________________________________________________________________

________________________________________________________________

________________________________________________________________

Display This Question:

If Q8 = Very high importance 4

Q8c You selected **"4 = Very high importance"**to:

***Intervention Delivery Schedule.*** *Report number of sessions, session duration, and session frequency including homework.*

Please provide your rationale for selecting this value. Include a reference to support your rationale, if possible.

________________________________________________________________

________________________________________________________________

________________________________________________________________

Display This Question:

If Q9 = Very high importance 4

Q9c You selected **"4 = Very high importance"**to:

***Interventionist.*** *Specify interventionist qualifications and/or credentials. Specify how many interventionists delivered study conditions.*

Please provide your rationale for selecting this value. Include a reference to support your rationale, if possible.

_____________________________________________________________

________________________________________________________________

Display This Question:

If Q10 = Very high importance 4

Q10c You selected **"4 = Very high importance"**to:

***Treatment Fidelity.*** *Describe strategies used to ensure that treatment and/or control conditions were delivered as intended (e.g., interventionist training, manualized protocols, intervention monitoring).*

Please provide your rationale for selecting this value. Include a reference to support your rationale, if possible.

________________________________________________________________

________________________________________________________________

________________________________________________________________

Display This Question:

If Q11 = Very high importance 4

Q11c You selected **"4 = Very high importance"**to:

***Setting.*** *Describe where the intervention was delivered; include location, privacy level, and ambient sound.*

Please provide your rationale for selecting this value. Include a reference to support your rationale, if possible.

________________________________________________________________

________________________________________________________________

________________________________________________________________

Display This Question:

If Q12 = Very high importance 4

Q12c You selected **"4 = Very high importance"**to:

***Unit of Delivery.*** *Specify whether interventions were delivered to individuals or groups of individuals, including the size of the group.*

Please provide your rationale for selecting this value. Include a reference to support your rationale, if possible.

________________________________________________________________

________________________________________________________________

________________________________________________________________

Q13 Thank you for sharing your input.

Are there additional criteria that you believe should be reported when describing music interventions in published research? If you have a reference to support your recommendation, please include that as well. *If none, please leave blank.*

________________________________________________________________

________________________________________________________________

________________________________________________________________

PAGE BREAK

Q14 Finally, please provide any additional comments that you would like to share about your responses or of this review. *If none, please leave blank.*

________________________________________________________________

________________________________________________________________

________________________________________________________________

**Supplemental Material**

**Round One Survey Results**

Panel members received this report in advance of the Round One Expert Panel meeting. We have removed representative statements from the supplemental materials to further ensure respondent anonymity.

**Introduction:**

Agreement Threshold: > 80% rate item as having “High” or “Very High” Importance. Items that met the threshold for importance are highlighted in green; items that did not meet the threshold are highlighted in red.

Comments: Common themes were identified by two independent reviewers, then discussed to reach consensus. We include common themes and representative statements for each item.

Note about Anonymity: Data received from the Indiana University Center for Survey Research was provided to us in aggregate form. All comments were de-identified and not linked to numeric ratings to ensure anonymity.

| **Q1** Rate the importance of **Intervention Theory** on a scale of 1 to 4. Provide a rationale for the music selected; specify how qualities and delivery of the music are expected to impact targeted outcomes. | | | | | |
| --- | --- | --- | --- | --- | --- |
|  | | Frequency | Percent | Valid Percent | Cumul Valid Percent |
| Valid | 1,2 Limited importance, Moderate importance | 6 | 9.2 | 9.7 | 9.7 |
|  | 3 High importance | 22 | 33.8 | 35.5 | 45.2 |
|  | 4 Very high importance | 34 | 52.3 | 54.8 | 100.0 |
|  | Total | 62 | 95.4 | 100.0 |  |
| Missing | Not answered | 3 | 4.6 |  |  |
| Total | | 65 | 100.0 |  |  |
| Agreement Threshold | 3 High + 4 Very High | 56 | 90.0 |  |  |
| Original Item A: Intervention Theory. Provide a rationale for the music selected; specify how qualities and delivery of the music are expected to impact targeted outcomes.  **Q1 Comments:**   - Current item language suggests use of music intervention theories and this may be too narrow. - Comments point to the need for refined wording, as rationale for MBI may be grounded in theory and/or scientific evidence. | | | | | |
| **Q2** Rate the importance of the **Intervention Content** on a scale of 1 to 4. Provide precise details of the music intervention and, when applicable, descriptions of procedures for tailoring interventions to individual participants. | | | | | |
|  | | Frequency | Percent | Valid Percent | Cumul Valid Percent |
| Valid | 2 Moderate importance; 3 High importance | 17 | 29.2 | 30.2 | 30.2 |
|  | 4 Very high importance | 44 | 67.7 | 69.8 | 100.0 |
|  | Total | 63 | 96.9 | 100.0 |  |
| Missing | Not answered | 2 | 3.1 |  |  |
| Total | | 65 | 100.0 |  |  |
| Agreement Threshold | 3 High + 4 Very High | 61 | 97.0 |  |  |
| Original Item B: Intervention Content. Provide precise details of the music intervention and, when applicable, descriptions of procedures for tailoring the intervention to participants.  **Q2 Comments:**   - This item is a section header. Because the order of reporting items was randomized, the survey respondents did not have context for this being a broader header that is followed by more specific items. This was evident in their comments. - Comments pointed to the need to refine wording. | | | | | |
| **Q3** Rate the importance of the **Intervention Content – Person Selecting the Music** on a scale of 1 to 4. Specify who selected the music: (1) pre-selected by investigators, (2) participant selected from limited set, (3) participant selected from own collections, or (4) tailored based on patient assessment. | | | | | |
|  | | Frequency | Percent | Valid Percent | Cumul Valid Percent |
| Valid | 2 Moderate importance | 5 | 7.7 | 8.1 | 8.1 |
|  | 3 High importance | 16 | 24.6 | 25.8 | 33.9 |
|  | 4 Very high importance | 41 | 63.1 | 66.1 | 100.0 |
|  | Total | 62 | 95.4 | 100.0 |  |
| Missing | Not answered | 3 | 4.6 |  |  |
| Total | | 65 | 100.0 |  |  |
| Agreement Threshold | 3 High + 4 Very High | 57 | 92.0 |  |  |
| Original Item B1: Person Selecting the Music. Specify who selected the music: (1) pre-selected by investigators, (2) participant selected from limited set, (3) participant selected from own collections, or (4) tailored based on patient assessment.  **Q3 Comments:**   - Comments point to the need for revised language and removal of specific options which are limited in scope and can become dated. | | | | | |
| **Q4** Rate the importance of the **Intervention Content – Music** on a scale of 1 to 4. When using published music, provide reference for sheet music or sound recording. When using improvised or original music, describe the music’s overall structure (i.e., form, elements, instruments, etc.). | | | | | |
|  | | Frequency | Percent | Valid Percent | Cumul Valid Percent |
| Valid | 1,2 Limited importance, Moderate importance | 14 | 21.5 | 22.2 | 22.2 |
|  | 3 High importance | 28 | 43.1 | 44.4 | 66.7 |
|  | 4 Very high importance | 21 | 32.3 | 33.3 | 100.0 |
|  | Total | 63 | 96.9 | 100.0 |  |
| Missing | Not answered | 2 | 3.1 |  |  |
| Total | | 65 | 100.0 |  |  |
| Agreement Threshold | 3 High + 4 Very High | 49 | 78.0 |  |  |
| Original Item B2: Music. When using published music, provide reference for sheet music or sound recording. When using improvised or original music, describe the music’s overall structure (i.e., form, elements, instruments, etc).  **Q4 Comments:**   - Comments point to the need for revised language. - Level of detail and description of the music will depend on subject and focus of the research (what was used vs. music characteristics/compositional features). This is also part of the theory and/or scientific rationale. | | | | | |
| **Q5** Rate the importance of the **Intervention Content – Music Delivery Method (Live or Recorded)** on a scale of 1 to 4. When using live music, specify who delivered the music and the size of the performance group (e.g., interventionist only, interventionist and participant). When using recorded music, specify placement of playback equipment and the use of headphones vs. speakers. Specify who determined/controlled volume (e.g., interventionist, participant). Specify decibel level of music delivered and/or use of volume controls to limit decibels. | | | | | |
|  | | Frequency | Percent | Valid Percent | Cumul Valid Percent |
| Valid | 2 Moderate importance | 8 | 12.3 | 13.1 | 13.1 |
|  | 3 High importance | 23 | 35.4 | 37.7 | 50.8 |
|  | 4 Very high importance | 30 | 46.2 | 49.2 | 100.0 |
|  | Total | 61 | 93.8 | 100.0 |  |
| Missing | Not answered | 4 | 6.2 |  |  |
| Total | | 65 | 100.0 |  |  |
| Agreement Threshold | 3 High + 4 Very High | 53 | 86.0 |  |  |
| Original Item B3: Music Delivery Method (live or recorded). When using live music, specify who delivered the music and the size of the performance group (e.g., interventionist only, interventionist and participant). When using recorded music, specify placement of playback equipment and the use of headphones vs. speakers. Specify who determined/controlled volume (e.g., interventionist, participant). Specify decibel level of music delivered and/or use of volume controls to limit decibels.  **Q5 Comments:**   - Overall comments suggest denoting live vs. recorded is important; however, details may be too granular and specific to only some populations and/or settings. - Size of the performance group moves into social aspects of the music experience. | | | | | |
| **Q6** Rate the importance of the **Intervention Content – Intervention Materials** on a scale of 1 to 4. Specify music and non-music materials. | | | | | |
|  | | Frequency | Percent | Valid Percent | Cumul Valid Percent |
| Valid | 2 Moderate importance | 13 | 20.0 | 20.3 | 20.3 |
|  | 3 High importance | 21 | 32.3 | 32.8 | 53.1 |
|  | 4 Very high importance | 30 | 46.2 | 46.9 | 100.0 |
|  | Total | 64 | 98.5 | 100.0 |  |
| Missing | Not answered | 1 | 1.5 |  |  |
| Total | | 65 | 100.0 |  |  |
| Agreement Threshold | 3 High + 4 Very High | 41 | 64.0 |  |  |
| Original Item B4: Intervention Materials. Specify music and/or non-music materials.  **Q6 Comments:**   - Comments point to the need for revised language, need for emphasis on delivery, and overlap with “Item B2: Music”. | | | | | |
| **Q7** Rate the importance of the **Intervention Content – Intervention Strategies** on a scale of 1 to 4. Describe music-based intervention strategies under investigation (e.g., music listening, songwriting, improvisation, lyric analysis, rhythmic auditory stimulation, etc.) | | | | | |
|  | | Frequency | Percent | Valid Percent | Cumul Valid Percent |
| Valid | 2,3 Moderate importance, High importance | 8 | 12.3 | 12.9 | 12.9 |
|  | 4 Very high importance | 54 | 83.1 | 87.1 | 100.0 |
|  | Total | 62 | 95.4 | 100.0 |  |
| Missing | Not answered | 3 | 4.6 |  |  |
| Total | | 65 | 100.0 |  |  |
| Agreement Threshold | 3 High + 4 Very High | 60 | 97.0 |  |  |
| Original Item B5: Intervention Strategies. Describe music-based intervention strategies under investigation (e.g., music listening, songwriting, improvisation, lyric analysis, rhythmic auditory stimulation, etc.)  **Q7 Comments:**   - Phrase “intervention strategies” is problematic and non-specific. Emphasis needs to be on providing detailed description of the intervention rather than a conceptual label. | | | | | |
| **Q8** Rate the importance of the **Intervention Delivery Schedule** on a scale of 1 to 4. Report number of sessions, session duration, and session frequency including homework. | | | | | |
|  | | Frequency | Percent | Valid Percent | Cumul Valid Percent |
| Valid | 2 Moderate importance | 3 | 4.6 | 4.8 | 4.8 |
|  | 3 High importance | 7 | 10.8 | 11.1 | 15.9 |
|  | 4 Very high importance | 53 | 81.5 | 84.1 | 100.0 |
|  | Total | 63 | 96.9 | 100.0 |  |
| Missing | Not answered | 2 | 3.1 |  |  |
| Total | | 65 | 100.0 |  |  |
| Agreement Threshold | 3 High + 4 Very High | 60 | 95.0 |  |  |
| Original Item C: Intervention Delivery Schedule. Report number of sessions, session duration, and session frequency including homework.  **Q8 Comments:**   - Comments centered on the need for revised language/terminology. | | | | | |
| **Q9** Rate the importance of the **Interventionist** on a scale of 1 to 4. Specify interventionist qualifications and/or credentials. Specify how many interventionists delivered study conditions. | | | | | |
|  | | Frequency | Percent | Valid Percent | Cumul Valid Percent |
| Valid | 2 Moderate importance | 5 | 7.7 | 8.2 | 8.2 |
|  | 3 High importance | 22 | 33.8 | 36.1 | 44.3 |
|  | 4 Very high importance | 34 | 52.3 | 55.7 | 100.0 |
|  | Total | 61 | 93.8 | 100.0 |  |
| Missing | Not answered | 4 | 6.2 |  |  |
| Total | | 65 | 100.0 |  |  |
| Agreement Threshold | 3 High + 4 Very High | 56 | 92.0 |  |  |
| Original Item D: Interventionist. Specify interventionist qualifications and/or credentials. Specify how many interventionists delivered study conditions.  **Q9 Comments:**   - Comments centered on adding information to capture range of experience and training more fully, and a suggestion to specify if one or more authors served as interventionist(s). | | | | | |
| **Q10** Rate the importance of the **Treatment Fidelity** on a scale of 1 to 4. Describe strategies used to ensure that treatment and/or control conditions were delivered as intended (e.g., interventionist training, manualized protocols, intervention monitoring). | | | | | |
|  | | Frequency | Percent | Valid Percent | Cumul Valid Percent |
| Valid | 2 Moderate importance | 3 | 4.6 | 4.7 | 4.7 |
|  | 3 High importance | 23 | 35.4 | 35.9 | 40.6 |
|  | 4 Very high importance | 38 | 58.5 | 59.4 | 100.0 |
|  | Total | 64 | 98.5 | 100.0 |  |
| Missing | Not answered | 1 | 1.5 |  |  |
| Total | | 65 | 100.0 |  |  |
| Agreement Threshold | 3 High + 4 Very High | 61 | 95.0 |  |  |
| Original Item E: Treatment Fidelity. Describe strategies used to ensure that treatment and/or control conditions were delivered as intended (e.g., interventionist training, manualized protocols, intervention monitoring).  **Q10 Comments:**   - Comments centered on clarifying item language. | | | | | |
| **Q11** Rate the importance of the **Setting** on a scale of 1 to 4. Describe where the intervention was delivered; include location, privacy level, and ambient sound. | | | | | |
|  | | Frequency | Percent | Valid Percent | Cumul Valid Percent |
| Valid | 1,2 Limited importance, Moderate importance | 16 | 24.6 | 25.4 | 25.4 |
|  | 3 High importance | 26 | 40.0 | 41.3 | 66.7 |
|  | 4 Very high importance | 21 | 32.3 | 33.3 | 100.0 |
|  | Total | 63 | 96.9 | 100.0 |  |
| Missing | Not answered | 2 | 3.1 |  |  |
| Total | | 65 | 100.0 |  |  |
| Agreement Threshold | 3 High + 4 Very High | 47 | 75.0 |  |  |
| Original Item F: Setting. Describe where the intervention was delivered: include location, privacy level, and ambient sound.  **Q11 Comments:**   - Comments raised important question about whether a description of the physical space and characteristics of that space are central to the “intervention description” or the “experimental design” which is covered in methodological guidelines. | | | | | |
| **Q12** Rate the importance of the **Unit of Delivery** on a scale of 1 to 4. Specify whether interventions were delivered to individuals or groups of individuals, including the size of the group. | | | | | |
|  | | Frequency | Percent | Valid Percent | Cumul Valid Percent |
| Valid | 2 Moderate importance | 4 | 6.2 | 6.3 | 6.3 |
|  | 3 High importance | 16 | 24.6 | 25.0 | 31.3 |
|  | 4 Very high importance | 44 | 67.7 | 68.8 | 100.0 |
|  | Total | 64 | 98.5 | 100.0 |  |
| Missing | Not answered | 1 | 1.5 |  |  |
| Total | | 65 | 100.0 |  |  |
| Agreement Threshold | 3 High + 4 Very High | 60 | 94.0 |  |  |
| Original Item F: Unit of Delivery. Specify whether interventions were delivered to individuals or groups of individuals, including the size of the group.  **Q12 Comments:**   - Comments centered on terminology and the relative importance as a discrete reporting item. | | | | | |
