# Supplementary material for: Reporting Guidelines for Music-based Interventions: an update and validation study
Source: Front Psychol. 2025 Jun 2;16:1551920. doi: 10.3389/fpsyg.2025.1551920 (PMC12171218; doi:10.3389/fpsyg.2025.1551920)
Supplement: Supplementary file 3 [file Supplementary_file_3.docx]

**Supplemental Material**

**Appendix C: Round Two Survey & Round Two Survey Results**

**Reporting Guidelines for Music-based Interventions Questionnaire Summer 2023**

**Instructions:** This survey provides a side-by-side comparison of reporting checklist items. The far-left column shows the original checklist items. The far-right column shows revised checklist items. Revisions for each item focused on wording to improve clarity.

Please read each item (A-G) and select one of three options:

I prefer the **original** checklist wording.

I have a suggested edit (use the open text box below each item to provide revised wording). I prefer the **revised** checklist wording.

*Please select one response for each row.*

# Q1

| ORIGINAL | I prefer  **original** | I have a suggested edit | I prefer  **revised*** | REVISED |
| --- | --- | --- | --- | --- |
|  | (1) | (2) | (3) |  |
| **A: Intervention Theory** - Provide a rationale for the music selected; specify how qualities and delivery of the music are expected to impact targeted outcomes. | o | o | o | **A: Intervention Theory and/or Scientific Rationale**  - Provide rationale for the music and/or music experience(s). Specify how essential features of the music and/or music experience(s) are expected to influence targeted outcomes. |

**Respondent(s) who selected “I prefer revised” and included a suggested edit Q1a were assigned a (4) code in the dataset and labeled as “I prefer revised – comment.”*

Q1a If you have a suggested edit, please include it in the space below. *If none, please leave this space blank.*

**B: Intervention Content** – For Items B1-B5, describe the intervention with enough detail to support replication. When applicable, describe procedures for tailoring the intervention.

# Q2

| ORIGINAL | I prefer  **original*** | I have a suggested edit | I prefer  **revised** | REVISED |
| --- | --- | --- | --- | --- |
|  | (1) | (2) | (3) |  |
| **B.1: Person Selecting** |  |  |  |  |
| **the Music** - Specify who |  |  |  |  |
| selected the music: (1) |  |  |  |  |
| pre-selected by |  |  |  | **B.1: Person(s)** |
| investigator, (2) participant |  |  |  | **Selecting the Music** - |
| selected from limited set,  (3) participant selected from own collection, or (4) | o | o | o | Describe processes for  how music was selected and who was involved |
| tailored based on patient |  |  |  | in music selection. |
| assessment. |  |  |  |  |

**Respondent(s) who selected “I prefer original” and included a suggested edit to Q2a were assigned a (5) code in the dataset and labeled as “I prefer original – comment.”*

Q2a If you have a suggested edit, please include it in the space below. *If none, please leave this space blank.*

| ORIGINAL | I prefer  **original** | I have a suggested edit | I prefer  **revised** | REVISED |
| --- | --- | --- | --- | --- |
|  | (1) | (2) | (3) |  |
| **B.2: Music** - When using published music, provide reference for sheet music or sound recording. When using improvised or original music, describe the music’s overall structure (i.e., form, elements, instruments, etc). | o | o | o | **B.2: Music** - Specify key details about the music that may be relevant to specified outcomes of interest. Characteristics may include compositional features of the music (tempo, melody, harmony, rhythm, pitch, tonality, form, instrumentation), sound intensity or volume, lyrics, and/or how the music relates to the cultural heritage and ethnicity of participants. |
|  |  |  |  |  |

Q3a If you have a suggested edit, please include it in the space below. *If none, please leave this space blank.*

| ORIGINAL | I prefer  **original** | I have a suggested edit | I prefer  **revised** | REVISED |
| --- | --- | --- | --- | --- |
|  | (1) | (2) | (3) |  |
| **B.3: Music Delivery Method (Live or Recorded)** - When using live music, specify who delivered the music and the size of the performance group (e.g., interventionist only, interventionist and participant).  When using recorded music, specify placement of playback equipment and the use of headphones vs. speakers. Specify who determined/controlled volume (e.g., interventionist; participant. Specify decibel level of music delivered and/or use of volume controls to limit decibels). | o | o | o | **B.3: Music Delivery Method** - Provide details about how music was provided to or created with participants (e.g., live, recorded, computer generated). Include any details necessary for replication. This might include size of performing group, use of playback equipment, person controlling volume. |

Q4a If you have a suggested edit, please include it in the space below. *If none, please leave this space blank.*

| ORIGINAL | I prefer  **original** | I have a suggested edit | I prefer  **revised** | REVISED |
| --- | --- | --- | --- | --- |
|  | (1) | (2) | (3) |  |
|  |  |  |  | **B.4: Materials** - List all |
| **B.4: Intervention** |  |  |  | materials necessary for |
| **Materials -** Specify music and/or non-  music materials. | o | o | o | the music experience. Include music and non-  music equipment and |
|  |  |  |  | materials. |
|  |  |  |  |  |

Q5a If you have a suggested edit, please include it in the space below. *If none, please leave this space blank.*

| ORIGINAL | I prefer  **original** | I have a suggested edit | I prefer  **revised** | REVISED |
| --- | --- | --- | --- | --- |
|  | (1) | (2) | (3) |  |
| **B.5: Intervention** |  |  |  |  |
| **Strategies** |  |  |  |  |
| - Describe music- |  |  |  | **B.5: Intervention** |
| based intervention |  |  |  | **Strategies** - Describe the |
| strategies under |  |  |  | music intervention strategy |
| investigation (examples: music listening, songwriting, | o | o | o | or strategies being studied (e.g., music listening, improvisation, song writing, |
| improvisation, lyric |  |  |  | rhythmic auditory |
| analysis, rhythmic |  |  |  | stimulation, etc). |
| auditory stimulation, |  |  |  |  |
| etc). |  |  |  |  |
|  |  |  |  |  |

Q6a If you have a suggested edit, please include it in the space below. *If none, please leave this space blank.*

# PAGE BREAK

| ORIGINAL | I prefer  **original** | I have a suggested edit | I prefer  **revised** | REVISED |
| --- | --- | --- | --- | --- |
|  | (1) | (2) | (3) |  |
|  |  |  |  | **C: Intervention Dosage** - |
|  |  |  |  | Report session length, |
| **C: Intervention** |  |  |  | frequency (e.g., 3x/week), time |
| **Delivery Schedule** - |  |  |  | interval between sessions |
| Report number of sessions, session  duration, and session frequency including | o | o | o | (e.g., single day, three consecutive days), and  duration (e.g., over 4 weeks) including any work or |
| practice sessions. |  |  |  | experiences completed by |
|  |  |  |  | participants between |
|  |  |  |  | intervention sessions. |
|  |  |  |  |  |

Q7a If you have a suggested edit, please include it in the space below. *If none, please leave this space blank.*

| ORIGNAL | I prefer  **original** | I have a suggested edit | I prefer  **revised** | REVISED |
| --- | --- | --- | --- | --- |
|  | (1) | (2) | (3) |  |
| **D: Interventionist** - Specify interventionist qualifications and credentials. Specify how many interventionists deliver study conditions. | o | o | o | **D: Interventionist** - Specify interventionist qualifications, credentials, training, and/or experience. Indicate how many interventionists delivered the studied activity or experience. |

Q8a If you have a suggested edit, please include it in the space below. *If none, please leave this space blank.*

| ORIGINAL | I prefer  **original** | I have a suggested edit | I prefer  **revised*** | REVISED |
| --- | --- | --- | --- | --- |
|  | (1) | (2) | (3) |  |
| **E: Treatment Fidelity** - Describe strategies used to ensure that treatment and/or control conditions were delivered as intended (e.g., interventionist training, manualized protocols, and intervention monitoring). | o | o | o | **E: Treatment Fidelity** - Describe strategies and/or measures used to ensure that the music intervention was delivered and received as intended. |

**Respondent(s) who selected “I prefer revised” and included a suggested edit Q9a were assigned a (4) code in the dataset and labeled as “I prefer revised – comment.”*

Q9a If you have a suggested edit, please include it in the space below. *If none, please leave this space blank.*

|  | I prefer  **original** | I have a suggested edit | I prefer  **revised*** |  |
| --- | --- | --- | --- | --- |
|  | (1) | (2) | (3) |  |
|  | o | o | o | **F: Setting** - Describe where |
| **F: Setting** - Describe |  |  |  | the intervention was |
| where the intervention |  |  |  | delivered. Include location, |
| was delivered; include |  |  |  | privacy level, ambient |
| location, privacy level, |  |  |  | sound, and/or any other |
| and ambient sound. |  |  |  | factors that may affect |
|  |  |  |  | participants’ experience. |

**Respondent(s) who selected “I prefer revised” and included a suggested edit Q10a were assigned a (4) code in the dataset and labeled as “I prefer revised – comment.”*

Q10a If you have a suggested edit, please include it in the space below. *If none, please leave this space blank.*

| I prefer  **original** | | I have a suggested edit | I prefer  **revised*** |  |
| --- | --- | --- | --- | --- |
|  | (1) | (2) | (3) |  |
| **G: Unit of Delivery**  - Specify whether interventions were delivered to individuals or groups of individuals, including the size of the group. | o | o | o | **G: Individual, Dyadic, or Group Intervention** - Specify whether interventions were delivered to individuals, dyads, or groups of individuals. For group interventions, specify size of the group. |

**Respondent(s) who selected “I prefer revised” and included a suggested edit Q11a were assigned a (4) code in the dataset and labeled as “I prefer revised – comment.”*

Q11a If you have a suggested edit, please include it in the space below. *If none, please leave this space blank.*

**Supplemental Material**

**Round Two Survey Results**

For each item, we used a consensus threshold of >80% to determine preference for the original or the revised item. In addition, the panel used open-ended comments from survey respondents to determine if an item required further revision.

Panel members received this report in advance of the Round Two Expert Panel Meeting. Panelists also received summaries of respondent comments to improve item clarity, including verbatim suggestions. We have removed this information from the supplemental material to ensure respondent anonymity.

Green highlights indicates where the consensus threshold was met; yellow highlights indicate where an item did not meet the consensus threshold.

| **Q1 A: Intervention Theory / Intervention Theory and / or Scientific Rationale** | | | | | |
| --- | --- | --- | --- | --- | --- |
|  | | Frequency | Percent | Valid Percent | Cumulative Percent |
| Valid | I prefer original | 4 | 6.6 | 6.6 | 6.6 |
|  | I have a suggested edit | 7* | 11.5 | 11.5 | 18.0 |
|  | I prefer revised | 49 | 80.3 | 80.3 | 98.4 |
|  | I prefer revised - comment | 1 | 1.6 | 1.6 | 100.0 |
|  | Total | 61 | 100.0 | 100.0 |  |
| * 1 respondent selected "I have a suggested edit," but did not provide a suggested edit in Q1a. | | | | | |

**Summary/Guiding Questions:** Revised language met threshold of > 80%. However, we want to consider open-ended comments (n=7) to determine if additional edits would provide improved clarity.

**Section B. Intervention Content. For Items B1-B5, describe the music intervention with enough detail to support replication. When applicable, describe procedures for tailoring the intervention.**

| **Q2 B.1: Person Selecting the Music** | | | | | |
| --- | --- | --- | --- | --- | --- |
|  | | Frequency | Percent | Valid Percent | Cumulative Percent |
| Valid | I prefer original | 11 | 18.0 | 18.3 | 18.3 |
|  | I have a suggested edit | 10 | 16.4 | 16.7 | 35.0 |
|  | I prefer revised | 38 | 62.3 | 63.3 | 98.3 |
|  | I prefer original - comment | 1 | 1.6 | 1.7 | 100.0 |
|  | Total | 60 | 98.4 | 100.0 |  |
| Missing | Seen but unanswered | 1 | 1.6 |  |  |
| Total | | 61 | 100.0 |  |  |

**Summary/Guiding Questions:** Revised language did not meet threshold of > 80%. Please consider open-ended comments (n=11) to identify any additional edits. Emphasis is on improved clarity.

| **Q3 B.2: Music** | | | | | |
| --- | --- | --- | --- | --- | --- |
|  | | Frequency | Percent | Valid Percent | Cumulative Percent |
| Valid | I prefer original | 2 | 3.3 | 3.3 | 3.3 |
|  | I have a suggested edit | 14 | 23.0 | 23.0 | 26.2 |
|  | I prefer revised | 45 | 73.8 | 73.8 | 100.0 |
|  | Total | 61 | 100.0 | 100.0 |  |

**Summary/Guiding Questions:** Revised language did not meet threshold of > 80%. Please consider open-ended comments (n=14) to identify any additional edits. Emphasis is on improved clarity.

| **Q4 B.3: Music Delivery Method (Live or Recorded)** | | | | | |
| --- | --- | --- | --- | --- | --- |
|  | | Frequency | Percent | Valid Percent | Cumulative Percent |
| Valid | I prefer original | 6 | 9.8 | 9.8 | 9.8 |
|  | I have a suggested edit | 6 | 9.8 | 9.8 | 19.7 |
|  | I prefer revised | 49 | 80.3 | 80.3 | 100.0 |
|  | Total | 61 | 100.0 | 100.0 |  |

**Summary/Guiding Questions:** Revised language met our threshold of > 80%. Please consider open-ended comments (n=6) to determine any additional edits. Emphasis is on improved clarity.

| **Q5 B.4: Intervention Materials / Materials** | | | | | |
| --- | --- | --- | --- | --- | --- |
|  | | Frequency | Percent | Valid Percent | Cumulative Percent |
| Valid | I prefer original | 1 | 1.6 | 1.6 | 1.6 |
|  | I have a suggested edit | 3 | 4.9 | 4.9 | 6.6 |
|  | I prefer revised | 57 | 93.4 | 93.4 | 100.0 |
|  | Total | 61 | 100.0 | 100.0 |  |

**Summary/Guiding Questions:** Revised language met our threshold of > 80%. Please consider open-ended comments (n=3) to determine any additional edits. Emphasis is on improved clarity.

| **Q6 B.5: Intervention Strategies** | | | | | |
| --- | --- | --- | --- | --- | --- |
|  | | Frequency | Percent | Valid Percent | Cumulative Percent |
| Valid | I prefer original | 6 | 9.8 | 9.8 | 9.8 |
|  | I have a suggested edit | 5 | 8.2 | 8.2 | 18.0 |
|  | I prefer revised | 50 | 82.0 | 82.0 | 100.0 |
|  | Total | 61 | 100.0 | 100.0 |  |

**Summary/Guiding Questions:**  Revised language met our threshold of > 80%.  Please consider open-ended comments (n=5) to determine any additional edits. Emphasis is on improved clarity.

| **Q7 C: Intervention Delivery Schedule / Intervention Dosage** | | | | | |
| --- | --- | --- | --- | --- | --- |
|  | | Frequency | Percent | Valid Percent | Cumulative Percent |
| Valid | I prefer original | 2 | 3.3 | 3.3 | 3.3 |
|  | I have a suggested edit | 5 | 8.2 | 8.2 | 11.5 |
|  | I prefer revised | 54 | 88.5 | 88.5 | 100.0 |
|  | Total | 61 | 100.0 | 100.0 |  |

**Summary/Guiding Questions:** Revised language met our threshold of > 80%. Please consider open-ended comments (n=5) to determine any additional edits. Emphasis is on improved clarity.

| **Q8 D: Interventionist** | | | | | |
| --- | --- | --- | --- | --- | --- |
|  | | Frequency | Percent | Valid Percent | Cumulative Percent |
| Valid | I prefer original | 1 | 1.6 | 1.6 | 1.6 |
|  | I have a suggested edit | 3 | 4.9 | 4.9 | 6.6 |
|  | I prefer revised | 57 | 93.4 | 93.4 | 100.0 |
|  | Total | 61 | 100.0 | 100.0 |  |

**Summary/Guiding Questions:** Revised language met our threshold of > 80%. Please consider open-ended comments (n=3) to determine any additional edits. Emphasis is on improved clarity.

| **Q9 E: Treatment Fidelity** | | | | | |
| --- | --- | --- | --- | --- | --- |
|  | | Frequency | Percent | Valid Percent | Cumulative Percent |
| Valid | I prefer original | 18 | 29.5 | 29.5 | 29.5 |
|  | I have a suggested edit | 9 | 14.8 | 14.8 | 44.3 |
|  | I prefer revised | 32 | 52.5 | 52.5 | 96.7 |
|  | I prefer revised - comment | 2 | 3.3 | 3.3 | 100.0 |
|  | Total | 61 | 100.0 | 100.0 |  |

**Summary/Guiding Questions:** Revised language did not meet threshold of > 80%. Please consider open-ended comments (n=11) to identify any additional edits. Emphasis is on improved clarity.

| **Q10 F: Setting** | | | | | |
| --- | --- | --- | --- | --- | --- |
|  | | Frequency | Percent | Valid Percent | Cumulative Percent |
| Valid | I prefer original | 3 | 4.9 | 4.9 | 4.9 |
|  | I have a suggested edit | 3 | 4.9 | 4.9 | 9.8 |
|  | I prefer revised | 54 | 88.5 | 88.5 | 98.4 |
|  | I prefer revised - comment | 1 | 1.6 | 1.6 | 100.0 |
|  | Total | 61 | 100.0 | 100.0 |  |

**Summary/Guiding Questions:** Revised language met our threshold of > 80%. Please consider open-ended comments (n=4) to determine any additional edits. Emphasis is on improved clarity.

| **Q11 G: Unit of Delivery / Individual, Dyadic, or Group Intervention** | | | | | |
| --- | --- | --- | --- | --- | --- |
|  | | Frequency | Percent | Valid Percent | Cumulative Percent |
| Valid | I prefer original | 3 | 4.9 | 5.0 | 5.0 |
|  | I have a suggested edit | 3 | 4.9 | 5.0 | 10.0 |
|  | I prefer revised | 53 | 86.9 | 88.3 | 98.3 |
|  | I prefer revised - comment | 1 | 1.6 | 1.7 | 100.0 |
|  | Total | 60 | 98.4 | 100.0 |  |
| Missing | Seen but unanswered | 1 | 1.6 |  |  |
| Total | | 61 | 100.0 |  |  |

**Summary/Guiding Questions:** Revised language met our threshold of > 80%. Please consider open-ended comments (n=4) to determine any additional edits. Emphasis is on improved clarity.
